# Structural determinants of *Neosartorya fischeri* antifungal protein (NFAP) for folding, stability and antifungal activity

**DOI:** 10.1038/s41598-017-02234-w

**Published:** 2017-05-16

**Authors:** László Galgóczy, Attila Borics, Máté Virágh, Hargita Ficze, Györgyi Váradi, Zoltán Kele, Florentine Marx

**Affiliations:** 1 Division of Molecular Biology, Biocenter, Medical University of Innsbruck, Innrain 80-82, 6020 Innsbruck, Austria; 2 Institute of Biochemistry, Biological Research Centre, Hungarian Academy of Sciences, Temesvári krt. 62, 6726 Szeged, Hungary; 3 Department of Microbiology, Faculty of Science and Informatics, University of Szeged, Közép fasor 52, 6726 Szeged, Hungary; 4 Department of Medical Chemistry, Faculty of Medicine, University of Szeged, Dóm square 8, 6720 Szeged, Hungary

## Abstract

The recent global challenges to prevent and treat fungal infections strongly demand for the development of new antifungal strategies. The structurally very similar cysteine-rich antifungal proteins from ascomycetes provide a feasible basis for designing new antifungal molecules. The main structural elements responsible for folding, stability and antifungal activity are not fully understood, although this is an essential prerequisite for rational protein design. In this study, we used the *Neosartorya fischeri* antifungal protein (NFAP) to investigate the role of the disulphide bridges, the hydrophobic core, and the N-terminal amino acids in the formation of a highly stable, folded, and antifungal active protein. NFAP and its mutants carrying cysteine deletion (NFAPΔC), hydrophobic core deletion (NFAPΔh), and N-terminal amino acids exchanges (NFAPΔN) were produced in *Pichia pastoris*. The recombinant NFAP showed the same features in structure, folding, stability and activity as the native protein. The data acquired with mass spectrometry, structural analyses and antifungal activity assays of NFAP and its mutants proved the importance of the disulphide bonding, the hydrophobic core and the correct N-terminus for folding, stability and full antifungal function. Our findings provide further support to the comprehensive understanding of the structure-function relationship in members of this protein group.

## Introduction

Recent global challenges to prevent and treat fungal infections for human welfare require the development of novel antifungal strategies against moulds^1^. The cysteine-rich antifungal proteins from filamentous ascomycetes (AFPs) provide a feasible basis for the rational design and development of new bio-pesticides, preservatives and drugs with improved activity, efficacy, fungal-selectivity, and low-cost production^2–5^. The members of this protein group show different antifungal spectra and mechanisms of action on opportunistic human, animal, plant and foodborne pathogenic filamentous fungi^6–9^, but interestingly, they exhibit remarkably similar β-barrel topology constituting five highly twisted antiparallel β-strands^10–14^. This structure is stabilized by three to four disulphide bridges, which provide stability against protease degradation, high temperatures and within a broad pH range^6^. In spite of the available experimental knowledge about their structure^10–14^, the main structural elements which coordinate the proper folding, and hence are responsible for the high-stability and antifungal activity have not been fully understood. However, distinction of these “essential” determinants from the other “non-essential” designable structural elements is a prerequisite for rational modifications to improve the activity and fungal-specificity of these proteins, which are strongly requested for novel antifungal strategies. The importance of disulphide bonds and the correct cleavage of the leader sequence from the N-terminus for structural integrity and antifungal activity have been already observed in this protein family^11, 12, 15, 16^.

The tertiary structure of AFPs is similar to the β-defensin(-like) peptides but they possess a hydrophobic core^12^. In the lack of a distinct hydrophobic core, the folded structure of β-defensins is stabilized by intramolecular disulphide bonds between cysteine residues and these also catalyse the proper folding of antimicrobial active structure^17, 18^. It is well-known that the hydrophobic core of a protein is responsible for stability, and coordinates the folding and structural formation^19–21^. Mutations in the hydrophobic core affect the stability and the correct protein folding^22^. Furthermore, the first five N-terminal residues of β-defensins facilitate the proper folding and the formation of canonical disulphide bond pattern^17^. Single N-terminal amino acid mutations or lack of the N-terminal residues causes structural rearrangements^23^ and/or changes in the antimicrobial specificity, efficacy or toxicity^24^.

The *Neosartorya fischeri* NRRL 181 isolate secretes a representative of AFPs, the *Neosartorya fischeri* antifungal protein (NFAP)^25^. NFAP is synthesised as a preproprotein which contains both a signal sequence for secretion and a prosequence that is removed before or during protein release into the supernatant^25^. We successfully used a *Pichia pastoris* heterologous expression system to produce high amounts of folded and active recombinant NFAP, which has the same antifungal activity as the native NFAP^9^.

In this study, we wanted to prove our assumption that distinct structural elements contribute to the secondary structure formation, proper folding, stability and antifungal activity of AFPs from ascomycetes like NFAP. To achieve this objective, we produced recombinant NFAP mutants in *P. pastoris* that vary in disulphide bridge formation (NFAPΔC), the hydrophobic core (NFAPΔh) and the N-terminus (NFAPΔN) (Table 1). In NFAPΔC the disulphide bridges, and in NFAPΔh the hydrophobic core were destroyed by amino acids substitutions. The NFAPΔN mutant carried three exchanges of the first five N-terminal amino acids of the mature protein. The folding property, structural stability, and antimicrobial activity of these NFAP mutants were investigated by thermal unfolding experiments, antifungal susceptibility tests, and functional tests and were compared with the wild-type NFAP.

**Table 1:** Amino acid sequence and *in silico* predicted physical and chemical properties of the mature NFAP and its mutants.

Protein	Molecular mass (Da)	Theoretical pI	Number of Cys	Net charge at pH = 7.0	GRAVY	Number of β-strands	Disulphide bond pattern*
LEYKGECFTKDNTCKYKIDGKTYLAKCPSAANTKCEKDGNKCTYDSYNRKVKCDFRH							
NFAP	6625.50	8.93	6	+5.0	−1.214	5	*abcabc*
LEYKGE**Y**FTKDNT**Y**KYKIDGKTYLAK**Y**PSAANTK**Y**EKDGNK**Y**TYDSYNRKVK**Y**DFRH							
NFAP∆C	6985.74	9.35	0	+5.1	−1.614	4	—
LE**S**KGECFTKDNTCK**S**K**S**DGKT**S**LAKCPSAANTKCEKDGNKCT**S**DSYNRKVKCDFRH							
NFAP∆h	6295.05	8.99	6	+5.0	−1.272	4	*bb*
**G**E**W**K**A**ECFTKDNTCKYKIDGKTYLAKCPSAANTKCEKDGNKCTYDSYNRKVKCDFRH							
NFAP∆N	6606.48	8.95	6	+5.0	−1.242	4	*bb*

Substitutions in the amino acid sequences are indicated with bold and underlined letters. ^*^The formation of disulphide bridges between C7-C35 and C27-C53 in NFAP∆h and NFAP∆N is impossible, as the distance between these cysteines is over the disulphide bonding limit, 2.3 Å^26^. GRAVY: grand average of hydropathy value; NFAP: *Neosartorya fischeri* NRRL 181 antifungal protein; NFAP∆C: cysteine deletion NFAP mutant; NFAP∆h: hydrophobic core deletion NFAP mutant; NFAP∆N: N-terminal amino acids exchanged NFAP mutant.

## Results

### Homology modelling suggested disrupted structure and disturbed folding of NFAPΔC, NFAPΔh and NFAPΔN

**Figure 1:**
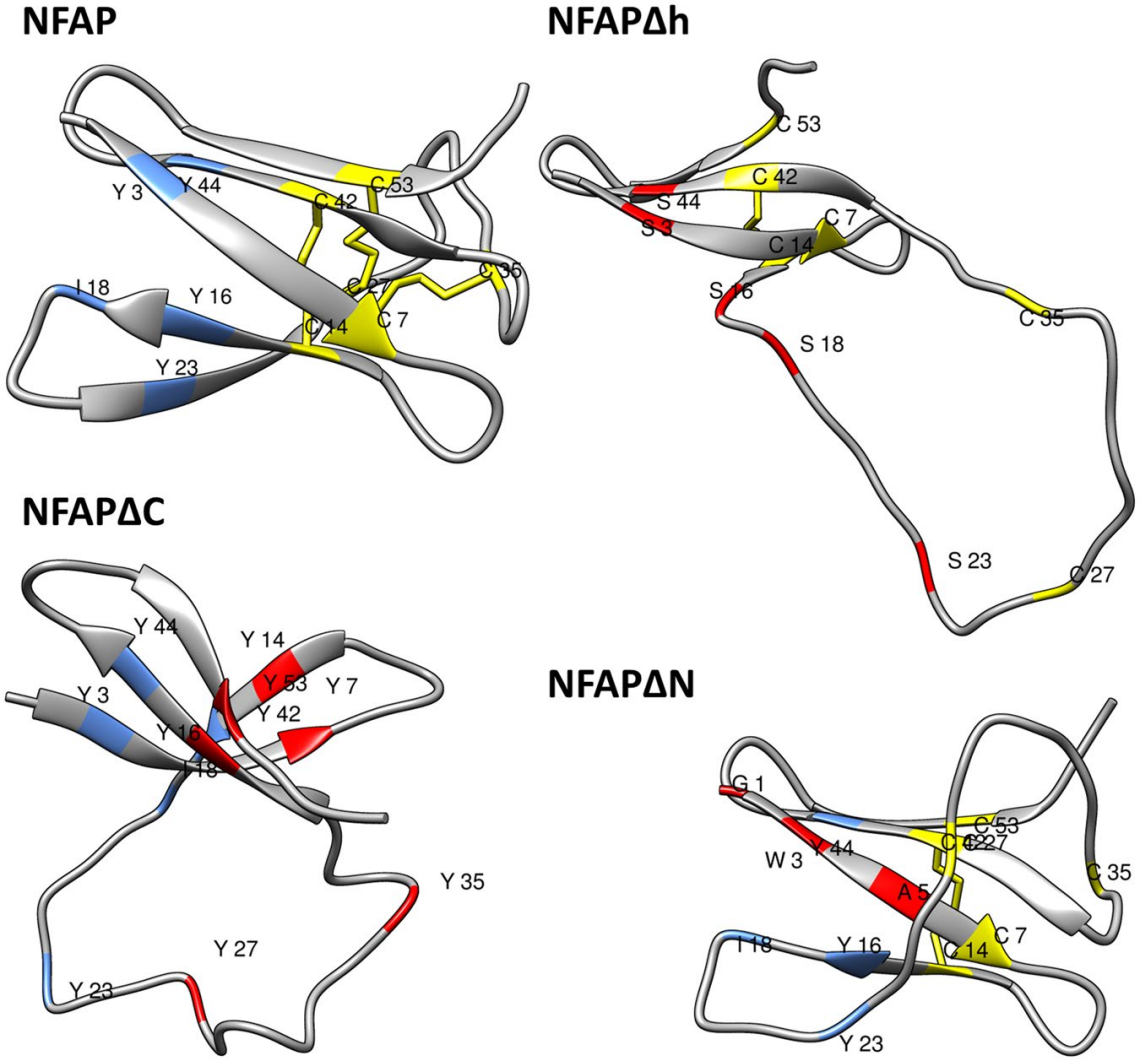
Predicted tertiary structure of NFAP, NFAPΔC, NFAPΔh, NFAPΔN. The hydrophobic core constituting amino acids are indicated by light blue. Cysteines and the possible disulphide bridges are marked with yellow and yellow line. Amino acid substitutions are indicated in red. The putative structures are highly reliable: Based on the Ramachandran plot analysis^37^, 94.5% and 5.5% of the residues are in the favoured and allowed regions at NFAP; and 98.2% and 1.8% at NFAPΔC, NFAPΔh, and NFAPΔN. The.pdb files of the structures are available in the Dataset 1–4. NFAP: *Neosartorya fischeri* NRRL 181 antifungal protein; NFAP∆C: cysteine deletion NFAP mutant; NFAP∆h: hydrophobic core deletion NFAP mutant; NFAP∆N: N-terminal amino acids exchanged NFAP mutant.

*In silico* homology modelling experiments can reveal the contribution of the disulphide bonding, the hydrophobic core and the N-terminus to formation of correct secondary structure and folding of NFAP. Hence, we analysed potential structural changes in NFAP when these structural elements are disturbed by amino acid replacement. The *in silico* predicted tertiary structure of NFAP contains five antiparallel β-strands (constituted by E2-C7, T13-K17, T22-K26, N40-D45, K50-D54) connected with four loops (F8-N12, I18-K21, C27-G39, S46-R49) (Fig. 1). This folded β-barrel structure is stabilized by three intramolecular disulphide bridges between C7-C35, C14-C42 and C27-C53 in an *abcabc* bonding pattern (Fig. 1). NFAP has an amphipathic surface (Supplementary Fig. S1a), alternating positively- and negatively-charged patches (Supplementary Fig. S1b) and a hydrophobic core constituted by Y3, Y16, I18, Y23, Y44 (Fig. 1). The disulphide-bonded six cysteines are well protected in the centre of this hydrophobic core. Replacements of all cysteines to tyrosines (C7Y, C14Y, C27Y, C35Y, C42Y, C53Y) abrogate the presence of the disulphide bridges (NFAPΔC, Table 1 and Fig. 1). Substitutions of tyrosines at the position of 3, 16, 23, 44 and the isoleucine at the position of 18 to serines (Y3S, Y16S, Y23S, Y44S, and I18S) destruct the hydrophobic core of the molecule (NFAPΔh, Table 1 and Fig. 1). The hydrophilicity of the N-terminal region can be increased by L1G, Y3W and G5A substitutions (NFAP∆N, Table 1 and Fig. 1), which results in a more hydrophobic N-terminal β-strand. However, these amino acid substitutions do not dramatically change the net charge and the grand average of hydropathy (GRAVY) value of the protein (Table 1), but based on the *in silico* homology modelling data they could impair the disulphide bridge formation^26^, secondary structure and folded state of NFAP (Fig. 1). The loss of one β-strand, the disturbed disulphide bonding and folding indicate the possibility of significant structural changes in all mutants (Fig. 1).

### *Pichia pastoris* KM71H produced NFAP and its mutants

To prove the structural and functional disruption by distinct amino acid substitutions, we produced NFAP and its mutants in *P. pastoris* KM71H (Supplementary Fig. S2). The average yield of purified NFAP, NFAPΔh, and NFAPΔN was 11.27 ± 4.65 mg/l (n = 3), 29.10 ± 0.77 mg/l (n = 3) and 7.62 ± 0.09 mg/l (n = 2), respectively. The NFAPΔC was degraded during its expression. Degradation of NFAPΔC became evident in sodium dodecyl sulphate-polyacrylamide gels (Supplementary Fig. S3) and did not allow a molar mass determination. To prove the presence of the degradation products of NFAPΔC in the culture broth of *P. pastoris* KM71H, the different molecular weight fractions of the ferment broth were subjected to tryptic digestion to allow the identification of defined peptide fragments originating from NFAPΔC by mass spectrometric (MS) analysis. These molecular mass data were then compared with the NFAPΔC sequence. Nine characteristic peptide fragments could be identified in the >10 kDa fraction which covered 74% of the NFAPΔC sequence (Supplementary Table S1). The identification of both the N- and C-terminal fragments of NFAPΔC proved that the protein was expressed in a correctly processed form and let us assume that it was degraded by extracellular proteases. The investigation of the <3 kDa fraction verified our assumption, that this fraction possibly contained the peptide fragments from the degraded NFAPΔC: We detected eight characteristic peptide fragments in it (Supplementary Table S1). The N-terminal YDFRH peptide fragment was not detectable in the fractions below <10 kDa, possibly due to its weak peak intensity. This result clearly indicates that NFAPΔC is not stable in the absence of the disulphide bridges, and is supposedly more prone to easy degradation by extracellular proteases.

### Mass spectrometry and RP-HPLC indicated truncations and structural variants of NFAPΔh and NFAPΔN

**Figure 2:**
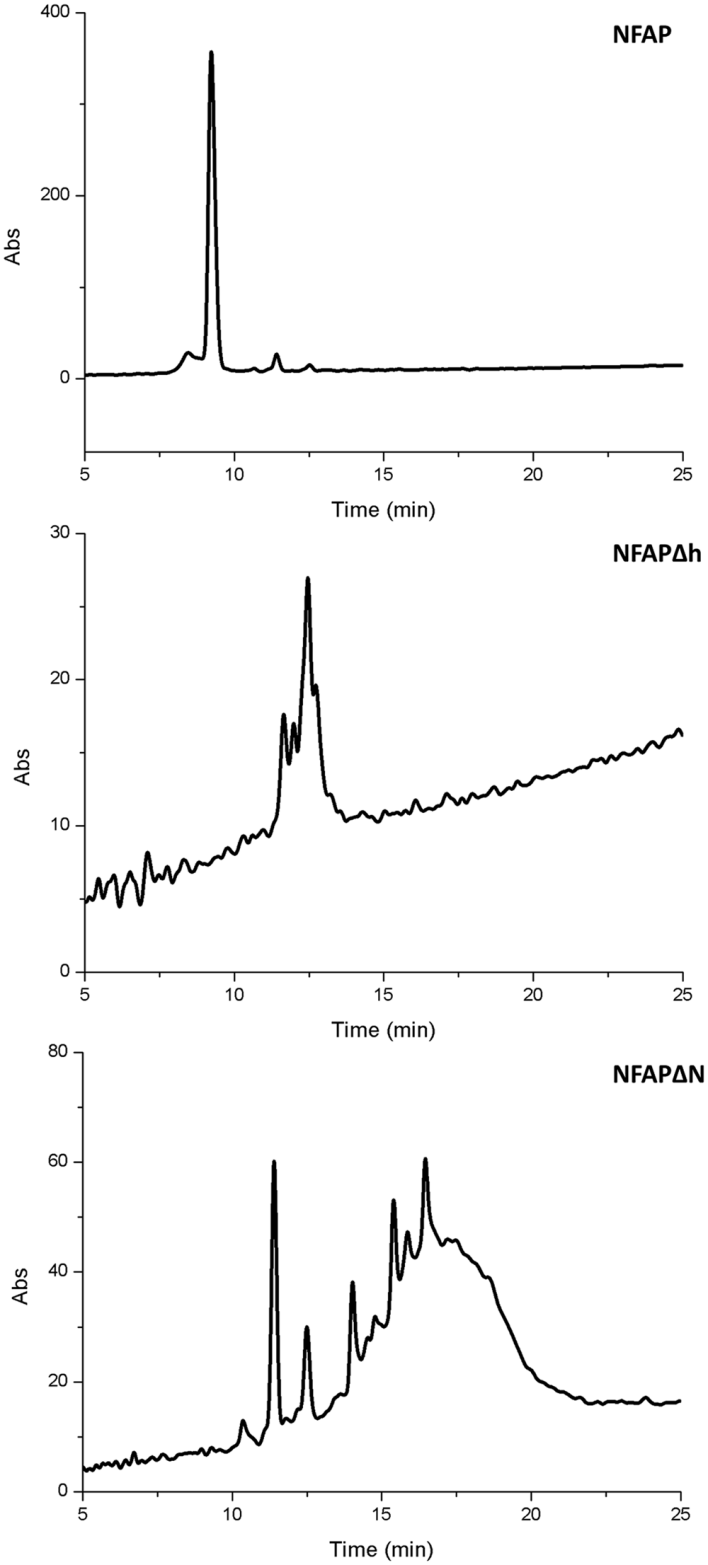
RP-HPLC chromatogram of recombinant NFAP, NFAPΔh, and NFAPΔN. Compared to NFAP, the NFAPΔh and NFAPΔN samples showed degradation and structural alterations, which were more prominent in NFAPΔN than in NFAPΔh. NFAP: recombinant *Neosartorya fischeri* NRRL 181 antifungal protein; NFAP∆h: hydrophobic core deletion NFAP mutant; NFAP∆N: N-terminal amino acids exchanged NFAP mutant.

NFAP, NFAPΔh, and NFAPΔN were identified by capillary electrophoresis electrospray ionization mass spectrometry (CE-ESI-MS). The calculated and measured monoisotopic molecular masses from independent expressions are listed in Table 2. The NFAP purified from four days cultivation broths of *P. pastoris* KM71H proved to be homogeneous and correctly maturated, and the measured molecular mass corresponded to the calculated mass of the protein that exhibits oxidized cysteines. This unambiguously indicated that all three disulphide bonds were formed (Table 2). In contrast, differently N- and C-terminal truncated, but disulphide bond-stabilized forms of NFAPΔh and NFAPΔN were present in the purified samples in addition to the full-length mature protein mutants (Table 2). Mass spectrum extraction analysis of the full-length NFAPΔh and NFAPΔN revealed that these NFAP mutants apparently showed different structures (Supplementary Fig. S4), and this could also be observed with their truncated variants (data not shown). This result indicates that NFAPΔh and NFAPΔN were expressed in unordered states before being stabilized by random disulphide bridges. The integrity of the mutant proteins was also examined by reversed-phase high performance liquid chromatography (RP-HPLC). While the NFAP sample proved to be intact (Fig. 2) and to exist in a single well-folded state, NFAPΔh and NFAPΔN samples were degraded and showed several different structures (Fig. 2). The structural alterations were more prominent in NFAPΔN than in NFAPΔh (Fig. 2). The RP-HPLC results further strengthen our assumption that these NFAP mutants are unordered and contain randomly matched disulphide bonds.

**Table 2:** Calculated and detected monoisotopic molecular masses (in the range of 5600–7400 Da) of the recombinantly expressed proteins that were purified from four days old *Pichia pastoris* KM71H supernatants.

Protein (calculated monoisotopic molecular mass with oxidized cysteine, Da)	Detected monoisotopic molecular mass by CE-ESI-MS (Da)	Protein variant	Number of disulphide bridges
NFAP (6615.10)	6615.07, 6615.08, 6615.17	*N-*NFAP*-C*	3
NFAPΔh (6284.92)	6284.90, 6284.98, 6284.99	*N-*NFAPΔh*-C*	3
	6171.90	*N-*(-L)NFAPΔh*-C*	3
	5827.63, 5827.71, 5827.73	*N-*(-LESK)NFAPΔh*-C*	3
	6147.83, 6147.92, 6147.93	*N-*NFAPΔh(-H)*-C*	3
	5991.72, 5991.81	*N-*NFAPΔh(-RH)*-C*	3
	5844.63, 5844.67	*N-*NFAPΔh(-FRH)*-C*	3
	5690.57, 5690.64	*N-*(-LESK)NFAPΔh(-H)*-C*	3
NFAPΔN (6596.07)	6596.04, 6596.09	*N-*NFAPΔN*-C*	3
	6095.80, 6095.87	*N-*(-GEWK)NFAPΔN*-C*	3
	6024.74	*N-*(-GEWKA)NFAPΔN*-C*	3
	5794.64	*N-*(-GEWKAEC)NFAPΔN*-C*	2
	6458.96, 6459.01	*N-*NFAPΔN(-H)*-C*	3
	6245.87	*N-*(-G)NFAPΔN(-RH)*-C*	3
	5958.73	*N-*(-GEWK)NFAPΔN(-H)*-C*	3

NFAP: recombinant *Neosartorya fischeri* NRRL 181 antifungal protein (n = 3); NFAP∆h: hydrophobic core deletion NFAP mutant (n = 3); NFAP∆N: N-terminal amino acids exchanged NFAP mutant (n = 2).

### ECD spectroscopy indicated unordered secondary structure of NFAPΔh and NFAPΔN

**Figure 3:**
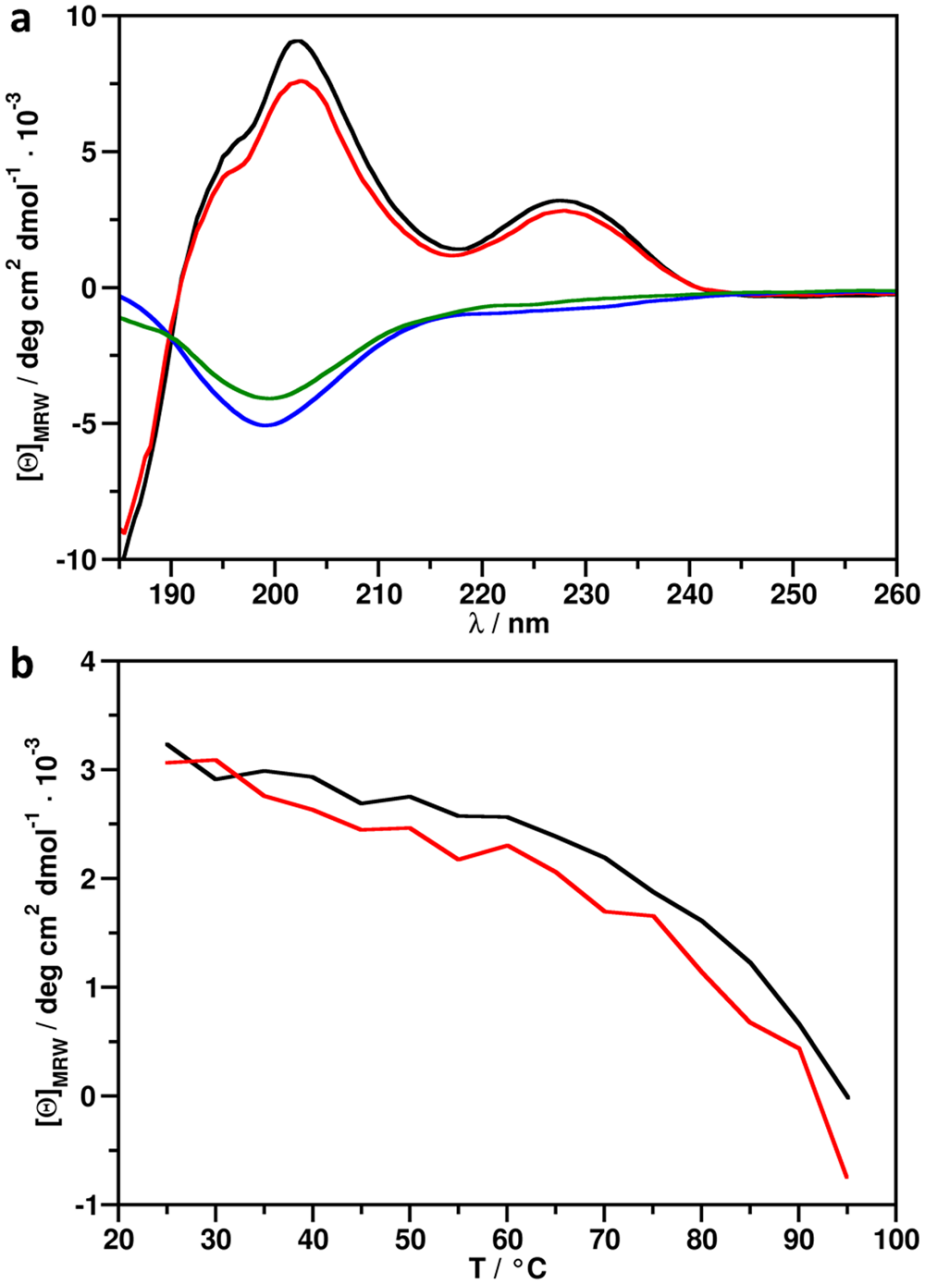
ECD spectra and thermal unfolding of NFAP, NFAPΔh, and NFAPΔN. (**a**) ECD spectra of native NFAP (black), recombinant NFAP (red), NFAPΔh (blue) and NFAPΔN (green) mutants recorded at 25 °C. (**b**) Thermal unfolding of native NFAP (black) and recombinant NFAP (red) followed by ECD spectroscopy at 228 nm. NFAP: native and recombinant *Neosartorya fischeri* NRRL 181 antifungal protein; NFAP∆h: hydrophobic core deletion NFAP mutant; NFAP∆N: N-terminal amino acids exchanged NFAP mutant.

The secondary structure of recombinant NFAP, NFAPΔh, and NFAPΔN and the thermal stability of recombinant NFAP were investigated by ECD spectroscopy and the results were compared with NFAP from the native producer *N. fischeri* NRRL 181^25^. ECD spectra of the native and recombinant NFAP were identical at 25 °C (Fig. 3a) indicating that their secondary structures are the same. These spectra had two maxima at 202 nm and 228 nm with a shoulder at 195 nm and a low intensity minimum centred at 217 nm. The maximum at 202 nm emerged from contributions from the spectral transitions of disulphide bridges. The maximum at 228 nm was mainly attributed to the disulphide bridges while the shoulder at 195 nm and the low intensity minimum at 217 nm indicated β-sheet conformation. Similar spectral features were reported previously for the homologous *Penicillium chrysogenum* antifungal protein (PAF)^14^ and other β-structured proteins which contain disulphide bridges^27^. Deconvolution of ECD spectra of native and recombinant NFAP revealed, that the difference between the secondary structure of the two proteins is not higher than 3% (Supplementary Table S2). In the case of NFAP, 3% accounts for less than two residues, therefore it can be assumed that the structures of the two proteins are essentially the same. Spectra of NFAPΔh and NFAPΔN reflected the complete loss of an ordered secondary structure and suggested the presence of various disulphide bond patterns. Thermal unfolding curves (Fig. 3b) indicated that unfolding was not complete in the 25 °C–95 °C temperature range. These curves did not present the usual sigmoidal shape; therefore, the exact determination of the melting temperature (T_m_) of the protein structure was not possible. Nevertheless, the native fold of NFAP appeared to be completely intact below 60 °C, which reflected the remarkable stability of this protein.

### NFAPΔh and NFAPΔN showed changes in the antifungal activity, thermal, pH and salt tolerance

**Figure 4:**
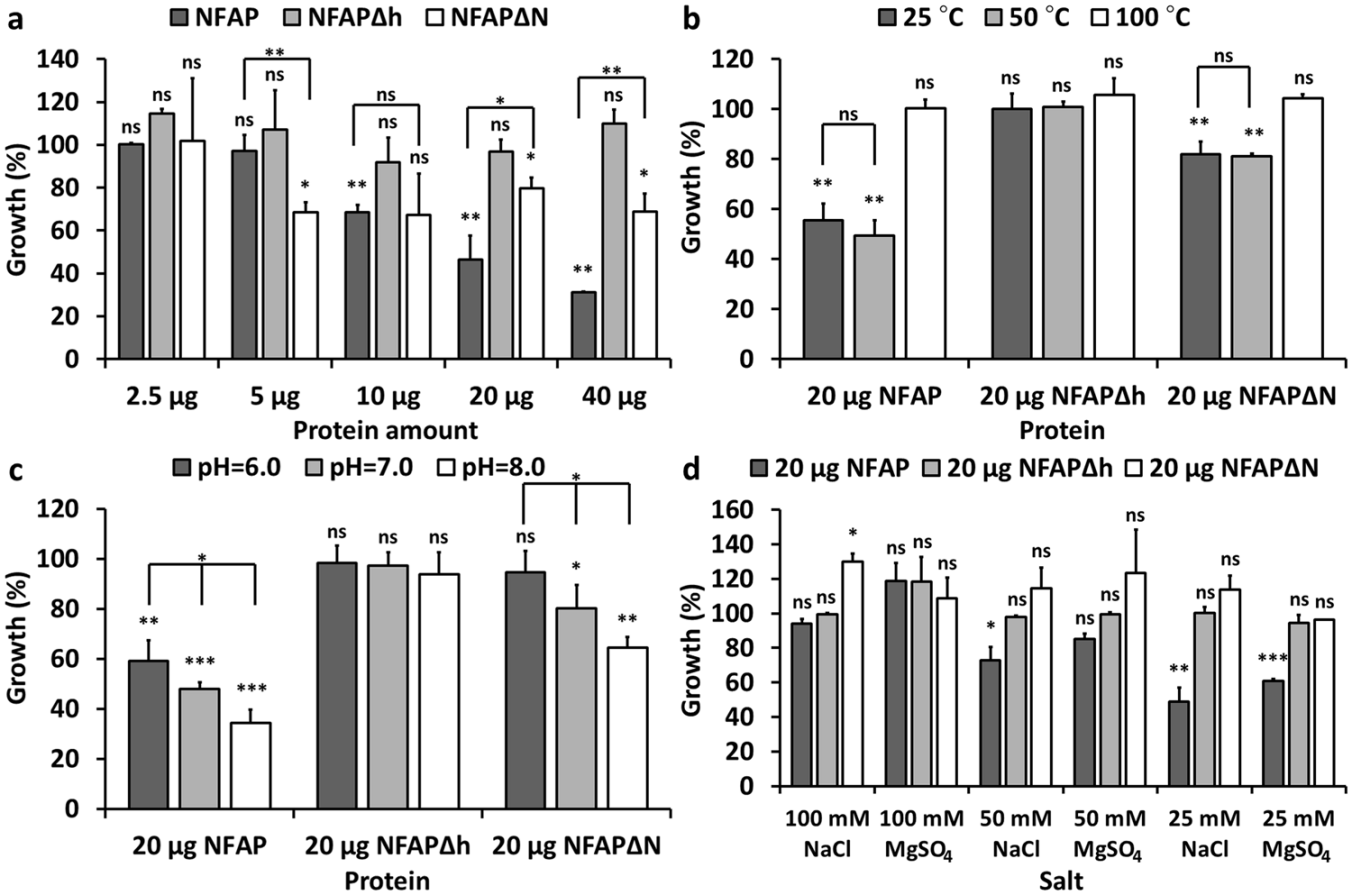
Antifungal activity of recombinant NFAP, NFAPΔh, NFAPΔN in broth microdilution tests. (**a**) Growth of *Aspergillus nidulans* FGSC A4 at pH 7.0 in the presence of 2.5–40 μg NFAP, NFAPΔh, and NFAPΔN; and with 20 μg of NFAP, NFAPΔh, and NFAPΔN after (**b**) heat-treatment at pH 7.0, (**c**) at different pH, and (**d**) in salt supplemented medium at pH 7.0. In all cases the untreated control cultures was referred to 100% growth. Significant differences (p-values) between the growth percentages were determined based on the comparison with the untreated control. When the growth percentages were compared in significance test, they are connected with line. ^***^p < 0.0001, ^**^p < 0.005, ^*^p < 0.05, ns: no significant difference. NFAP: recombinant *Neosartorya fischeri* NRRL 181 antifungal protein; NFAP∆h: hydrophobic core deletion NFAP mutant; NFAP∆N: N-terminal amino acids exchanged NFAP mutant.

The antifungal activity, thermal, pH and salt tolerance of the investigated proteins were tested in a broth microdilution assay using the NFAP-sensitive *Aspergillus nidulans* FGSC A4 as test organism. Compared to the ordered NFAP, the unordered NFAPΔh and NFAPΔN showed no or significantly reduced antifungal activity against *A. nidulans* FGSC A4, respectively (Fig. 4a). Surprisingly, five microgram NFAPΔN reduced the growth of the test organism to 68 ± 4.7% (Fig. 4a), whereas the same amount of NFAP proved to be inactive. A dose-dependent inhibitory activity was observed for NFAP, and in contrast to this, NFAPΔN reduced the growth with ca. 33%, independently of its applied amount (Fig. 4a). The difference between the antifungal activity of NFAP and NFAPΔN was significant at the applied amount of 5, 20 and 40 μg (Fig. 4a). NFAPΔN showed same tolerance characteristics to heat and pH as NFAP (Fig. 4b,c), but proved to be less salt-tolerant (Fig. 4d). Temperature treatment at 50 °C did not cause significant reduction in the antifungal activity of NFAP and NFAPΔN, but both proteins were inactive after treatment at 100 °C (Fig. 4b). NFAP and NFAPΔN were similarly pH sensitive (Fig. 4c). They showed the highest activity at slightly basic pH 8.0 and they were less active at slightly acidic pH 6.0 (Fig. 4c). Whilst 50–100 mM NaCl, and 25–100 mM MgSO_4_ impaired the antifungal activity of NFAP in a dose-dependent manner, presence of 25 mM NaCl had no influence (Fig. 4d). In contrast to this, NFAPΔN readily was inactivated by 25 mM NaCl or MgSO_4_. NFAPΔh was inactive under all conditions tested (Fig. 4a–d).

### NFAPΔh and NFAPΔN had no effects on hyphal morphology and physiology

Previously, we described that at sublethal concentrations NFAP affects the morphology of *A. nidulans* hyphae (hyperbranching and swollen hyphal tips, Supplementary Fig. S5a) as a consequence of disturbed actin distribution (Supplementary Fig. S5b) and chitin deposition (Supplementary Fig. S5c)^8^. Moreover, metabolic inactivation by NFAP was detected (Supplementary Fig. S5d)^8^. However, morphology (Supplementary Fig. S5a), actin distribution (Supplementary Fig. S5b), chitin deposition (Supplementary Fig. S5c) and metabolic activity (Supplementary Fig. S5c) of the *A. nidulans* hyphae were neither affected by NFAPΔh nor by NFAPΔN (Supplementary Fig. S5a–d).

## Discussion

Following previous observations from other cysteine-rich, β-structured antimicrobial peptides and proteins^17–24^, folding, stability and antifungal activity of NFAP was proposed here to be dependent of the correct disulphide bond pattern, the presence of a hydrophobic core and the correct N-terminal amino acid sequence. Previously we demonstrated that *P. pastoris* KM71H is able to produce folded, antifungal active NFAP, and its antifungal efficacy is comparable to the native protein^9^. In the present study ECD spectroscopic measurements proved that this recombinant NFAP has the same structural elements, folding and thermal stability as the native protein (Fig. 3a,b). Thus, any differences in structure and activity of the NFAP mutants generated in this study, reflect the possible role of disulphide bonding, hydrophobic core and N-terminal amino acid sequence for the function of the native NFAP.

*In silico* homology modelling was employed to visualize potential structural disruption induced by amino acid exchanges in the respective motifs of NFAP. The modelling estimated the possible impact of amino acid exchanges on the overall solution structure of the protein. The models suggest that all NFAP mutants exhibit four β-strands instead of the five β-strands present in native NFAP and less disulphide bonds. This could be an indication of strong perturbation of the ordered secondary structure. The drastic structural rearrangements of the NFAP mutants in the *in silico* homology model strongly indicated that the affected structural elements could have a deep impact in the protein folding. However, one must be aware of the fact that this method cannot predict if a protein is unordered or not. Thus, the homology modelling was helpful to investigate the importance of structural elements in the folding, but no evidence for the overall solution structure of a protein is given. The ECD spectroscopy provided an experimental insight into this aspect.

A loss of ordered structure was indicated by ECD spectroscopic measurements of NFAP mutants, however, it has to be further investigated how the amino acid exchanges interfere with the overall three-dimensional solution structure of NFAP. Clearly, we plan to approach this objective in the near future by replacing further amino acids in NFAP and analysing the impact on its actual structure at atomic resolution by nuclear magnetic resonance.

In this study, however, we performed the first important steps towards a better understanding of the NFAP structure-function relation. We could show that the lack of disulphide bonds render NFAPΔC highly sensitive to proteolytic degradation (Supplementary Fig. S3, Supplementary Table S1). The presence of all disulphide bonds and formation of the correct disulphide bonding pattern proved to be also important for the structural integrity and antifungal activity of other, NFAP-related antifungal proteins, the *Aspergillus giganteus* AFP^11^ and *P. chrysogenum* PAF^12, 16^. Considering this last observation and that NFAPΔC was totally degraded during expression, we conclude that the presence of all disulphide bonds in the correct pattern is indispensable for stability and full antifungal activity.

In this study, we observed for the first time that the hydrophobic core determines the folded and stable structure of NFAP. Substitution of neutral, hydrophobic amino acids (*i.e*. Y3, Y16, I18, Y23, Y44 at NFAP) to the neutral, hydrophilic serine in this region resulted in a mixture of differently truncated, unordered protein variants (NFAPΔh in Table 2 and Fig. 3a). Since neutral, hydrophobic amino acids are present at similar conserved positions in the primary structure of the so far isolated and characterized NFAP-related proteins (Supplementary Fig. S6), the universal role of the hydrophobic core in the proper protein folding of AFPs can be assumed. However, this hypothesis awaits further investigations.

NFAPΔN proved to be unstable (Table 2) and unordered (Fig. 3a) which indicates that the correct amino acid composition of the N-terminus of the mature protein is important for folding and stability. Information about the structural role of the first N-terminal amino acids of mature AFPs has not been reported so far; however, it was described that the antifungal activity of AFP is lost when it is not completely processed during fermentation and contains six additional amino acids at the N-terminus^15^. The role of the N-terminus in folding, stability and antimicrobial activity of defensins is also emphasised in the literature^17, 23, 24^.

The unordered NFAPΔh and NFAPΔN variants that lack amino acids at their N- and/or C-termini seemed to be more sensitive to extracellular protease degradation than NFAP. It is also possible that they could be differently processed during their maturation in the endoplasmic reticulum, the Golgi complex and the extracellular space. However, further investigation needs to prove these assumptions.

The disulphide bond-stabilized, folded protein form is required for the antifungal activity of AFP^11^ and PAF^16^, hence we were curious about the antifungal activity of the unordered NFAPΔh, and NFAPΔN mutants in comparison with the folded NFAP. NFAPΔh proved to be inactive in all susceptibility tests (Fig. 4a–d), and NFAPΔN showed a reduction in antifungal activity. These results clearly indicate that the ordered, folded protein structure is also essential for the full antifungal activity of this AFP- and PAF-related protein (Fig. 4a). Heat treatment experiments with NFAP further evidenced the importance of the folded structure in the antifungal activity. After treatment at 50 °C NFAP showed the same antifungal activity as the sample which was not heated (Fig. 4b), and based on the ECD spectroscopic measurements NFAP is still intact and retains its folded structure at this temperature (Fig. 2b). In contrast, after heat treatment at 100 °C, the protein became unfolded (Fig. 3b) and lost its antifungal activity (Fig. 4b).

Heat-, pH-, and salt-tolerances of NFAP (Fig. 4b–d) observed in this study parallel well with our previously observed published data with *Aspergillus niger* ^25^ or *A. nidulans* ^28^. The thermal- and pH-sensitivity of NFAPΔN was similar to NFAP. However, NFAPΔN proved to be more sensitive to the ion strength of the medium (Fig. 4d), which might be possibly the reason of its reduced activity. Interestingly, NFAP∆N did not cause morphological or physiological changes on hyphae in contrast to NFAP, but slightly inhibited the fungal growth in a dose-independent manner. This needs further investigations. Other studies with plant defensins^29^ and the NFAP-related antifungal protein PAF from *P. chrysogenum* ^30, 31^ reported that specific protein motifs exhibit distinct antifungal features. Therefore, the observed functional differences of the NFAP mutants reflect the importance of the mutated motifs for full integrity and optimal antifungal action.

The *in silico* analysis of the structure, the folding dynamics and the antifungal properties of NFAP and its mutants provided a detailed insight into the role of the disulphide bonds, the hydrophobic core and the N-terminal amino acids of the mature protein for proper folding, stability and antifungal activity. The results presented in this study provide further evidences to the comprehensive understanding of the structure-function relationship of other members of the AFP group, considering their structural similarities. This is an important prerequisite for their rational design to develop new protein-based antifungal strategies in the near future.

## Methods

### *In silico* predictions and homology modelling

The molecular weight, pI, GRAVY value, total net charge, and disulphide bridge pattern of proteins were calculated and predicted by ExPASy ProtParam tool^32^, Protein Calculator v3.4 server (The Scripps Research Institute; http://www.scripps.edu/~cdputnam/protcalc.html), and DISULFIND Cysteines Disulfide Bonding State and Connectivity Predictor server^33^, respectively. The experimentally determined NFAP-related *A. giganteus* antifungal protein tertiary structure (Protein Data Bank (PDB) code: 1afp) served as a template to model the structure of NFAP. Putative tertiary structure of NFAP was predicted *in silico* by MODELLER 9.9^34^, refined by ModRefiner^35^, and energy minimized and visualized with the UCSF Chimera software^36^. Residues in most favoured positions were validated by using the RAMPAGE server^37^. This *in silico* predicted tertiary structure of NFAP was used as a template to model the structure of NFAPΔC, NFAPΔh and NFAPΔN in the same way.

### Cloning, transformation and protein preparation

NFAP, NFAPΔC, NFAPΔh and NFAPΔN encoding cDNA were synthetized by GenScript USA Inc. (Piscataway, NJ, USA) considering the preferential codon usage of *P. pastoris*. The synthetic genes carried the restriction site of *Xho*I and the Kex2 signal cleavage site (CTC GAG AAA AGA) at their 5′-end, and the restriction site of *Xba*I (TCT AGA) at their 3′-end. They were cloned into *Xho*I-*Xba*I digested pPICZαA expression vector (Thermo Fisher Scientific, Waltham, MA, USA) and these constructs were used to transform *P. pastoris* KM71H (Thermo Fisher Scientific, Waltham, MA, USA) cells as previously described^9^. Heterologous protein expression in *P. pastoris* KM71H for four days and protein purification were performed on a CM-Sepharose column as reported^9^.

### Identification of produced proteins

Purified proteins were identified based on their molecular mass at the Protein Micro-Analysis Facility of Biocenter at Medical University of Innsbruck (Innsbruck, Austria). The protein samples were ZipTip enriched (EMD; Millipore, Billerica, MA, USA), dissolved in 100 mM acetic acid and analysed (180 nl/min flow rate) with capillary electrophoresis (CE)-ESI-MS (20 kV separation voltage, 10 psi pressure) on a CESI 8000 (AB Sciex, Framingham, MA, USA) coupled to a Q Exactive (Thermo Fisher Scientific, Waltham, MA, USA). Protein masses were determined by deconvolution using the integrated Xcalibur pXtract software (Thermo Fisher Scientific, Waltham, MA, USA).

The expression of NFAPΔC was verified by the identification of different peptide fragments from the degraded product. The NFAPΔC supernatant was separated into four different molecular weight fractions (>10 kDa, <10 kDa, 3–10 kDa and <3 kDa) by centrifugal ultrafiltration (Vivaspin 500 10,000 MWCO PES then 3,000 MWCO PES; Sartorius Stedim Biotech GmbH, Gottingen, Germany), before a mass spectrometric method was used to identify peptide fragments derived from NFAPΔC. This method was based on enzymatic digestion of the proteins present in each fraction and peptide mass mapping (Protein Prospector, MS-fit http://prospector.ucsf.edu/). For in solution protein digestion, 10 μl of protein solution containing 1 μg/μl protein was mixed with a buffer containing 25 mM ammonium bicarbonate, pH 8.0. The protein was subjected to enzymatic cleavage with 0.1 μg trypsin (Promega, Madison, WI, USA) solution (in 25 mM ammonium bicarbonate) overnight at 37 °C. Digested samples were analysed on a Waters NanoAcquity UPLC (Waters MS Technologies, Manchester, UK) system coupled with a Q Exactive Quadrupole-Orbitrap mass spectrometer (Thermo Fisher Scientific, Waltham, MA, USA). LC conditions were the followings: flow rate: 350 nl/min; eluent A: water with 0.1% (v/v) formic acid, eluent B: acetonitrile with 0.1%(v/v) formic acid; gradient: 40 min, 3–40% (v/v) B eluent; column: Waters BEH130 C18 75 lm/250 mm column with 1.7 μm particle size C18 packing (Waters Inc., Milford, MA, USA). Based on our previous experiences with the filtration of cysteine-rich and cationic antifungal proteins (and possibly their degradation products) showing high-affinity for the filter membrane material occlusion of the pores can occur, which inhibits the filtration of the proteins and bigger peptide fragments. Hence, the >10 kDa fraction was also analysed, although the molecular weights of the NFAPΔC peptides fragments were expected to be much below this cut-off.

### Investigation of structural alterations

Structural alterations of NFAP and its mutants were examined by RP-HPLC, using an Agilent 1100 Series liquid chromatograph (Agilent technologies, Little Falls, DE, USA) and a Phenomenex Jupiter C18 column (250 × 4.6 mm; 10 μm particle size; 300 Å pore size; Phenomenex, Torrance, CA, USA). Linear gradient elution was carried out with 0.1% TFA in water (eluent A) and 80% acetonitrile and 0.1% TFA is water (eluent B) from 15% to 40% (B) over 25 min at a flow rate of 1.0 ml/min.

### Electronic circular dichroism spectroscopy

Electronic circular dichroism (ECD) spectroscopic measurements of NFAP and the mixtures of unordered NFAPΔh, and NFAPΔN variants were performed in the 185–260 nm wavelength range using a Jasco-J815 spectropolarimeter (JASCO, Tokyo, Japan). Protein samples were presented in pure water at 0.1 mg/ml concentration and spectra were collected at 25 °C with a scan speed of 100 nm/s using a 0.1 cm pathlength quartz cuvette. The reported spectra are accumulations of 10 scans, from which the spectrum of pure water was subtracted. Acquisition of thermal unfolding curves of proteins was done by recording ellipticity as a function of temperature at 228 nm. The temperature was increased from 25 °C up to 95 °C in 5 °C increments, at a rate of 1 °C/min using a Peltier thermoelectronic controller (TE Technology, Traverse City, MI, USA). Measurements of ellipticities were taken at each temperature point after allowing the system to equilibrate for 1 min. Ellipticity data were corrected for protein concentration, which was determined based on the UV absorbance of aromatic and cysteine residues, following the protocol published by Greenfield, N. J.^38^ Secondary structural contributions were determined by the CDSSTR method from the ECD spectra of native and recombinant NFAP measured at 25 °C^39^.

### *In vitro* antifungal susceptibility tests

*In vitro* susceptibility tests were performed in a 96-well microtiter plate bioassay in the presence of increasing amount of proteins (2.5–40 μg) in SPEC medium at pH 7.0^25^ against the NFAP-sensitive model fungus *A. nidulans* strain FGSC A4 (Fungal Genetics Stock Center, Kansas, MO, USA) as described^8^. To investigate the salt, pH, and temperature sensitivity of the proteins (20 μg) the medium was supplemented with NaCl or MgSO_4_ (25–100 mM), or it was prepared in phosphate buffer (50 mM, pH 6.0–8.0), or exposed to different temperatures (25, 50, 100 °C) for 30 min. The flat-bottom plates were incubated for 48 hours at 37 °C without shaking, then after shaking for five seconds, the absorbance (OD_620_) were measured in well scanning mode with a microtiter plate reader (FLUOstar Omega, BMG Labtech, Ortenberg, Germany). Respective fresh media were used for background calibration. For calculation of the growth ability in presence of NFAP and the mixtures of unordered NFAPΔh, and NFAPΔN variants, the absorbance of the untreated control cultures (media without NFAP or its mutants) were referred to 100% growth. Susceptibility tests were prepared in triplicates and repeated three times.

### Investigation of the antifungal mechanism

The effect of NFAP and its mutants on the metabolic activity, actin distribution, and chitin content of *A. nidulans* FGSC A4 and *A. nidulans* GR5^40^ strains was investigated by means of 30 minutes-long exposure to sublethal protein concentrations (25 μg/ml) as described previously^8^.

### Microscopy

Cells were visualized by light and fluorescence microscopy (Carl Zeiss Axiolab LR 66238C; Zeiss, Oberkochen, Germany) and photographed by a microscope camera (Zeiss AxioCam ERc 5s; Zeiss, Oberkochen, Germany).

### Statistical analyses

Statistical analysis was performed using Microsoft Excel 2010 software (Microsoft, Edmond, WA, USA) or GraphPad Prism version 5.01 (GraphPad Software, San Diego, CA, USA). Two sample t-test or one-way ANOVA analysis of variance with Bonferroni’s multiple comparison posttest was used.

## Electronic supplementary material

Supplementary Information

Dataset 1

Dataset 2

Dataset 3

Dataset 4
