# Supplementary material for: Structural determinants of Neosartorya fischeri antifungal protein (NFAP) for folding, stability and antifungal activity
Source: Sci Rep. 2017 May 16;7:1963. doi: 10.1038/s41598-017-02234-w (PMC5434006; doi:10.1038/s41598-017-02234-w)
Supplement: Supplementary file 1 — Supplementary Information [file 41598_2017_2234_MOESM1_ESM.pdf]

Scientific Reports - Supplementary Information

Structural determinants of *Neosartorya fischeri* antifungal protein (NFAP) for folding, stability and antifungal activity

László Galgóczy[1,*], Attila Borics[2], Máté Virágh[3], Hargita Ficze[3], Györgyi Váradi[4], Zoltán Kele[4] & Florentine Marx[1]

[1]Division of Molecular Biology, Biocenter, Medical University of Innsbruck, Innrain 80-82, 6020 Innsbruck, Austria.
[2]Institute of Biochemistry, Biological Research Centre, Hungarian Academy of Sciences, Temesvári krt. 62, 6726 Szeged, Hungary.
[3]Department of Microbiology, Faculty of Science and Informatics, University of Szeged, Közép fasor 5, 6726 Szeged, Hungary.
[4]Department of Medical Chemistry, Faculty of Medicine, University of Szeged, Dóm square 8, 6720 Szeged, Hungary.

[*]Correspondence and requests for materials should be addressed to L.G. (e-mail: galgoczi@gmail.com)

Table S1: Identified peptide fragments of degraded NFAPΔC.

m/z submitted	MH^+ equivalent	MH^+ matched	Intensity	Delta ppm	Peptide combination	Elemental composition	Modifications	Start	End	Missed cleavages	Sequence
362.2056+2	723.4039	723.4036	3743166.2	0.419	0(0B)	C33 H55 N8 O10		16	21	1	(K)YKIDGK(T)
369.1719+2	737.3366	737.3365	13720476.0	0.0326	0(0B)	C34 H45 N10 O9		53	57	1	(K)YDFRH(-)
426.2164+2	851.4256	851.425	3405855.8	-0.196	0(0B)	C37 H59 N10 O13		27	34	0	(K)YPSAANTK(Y)
466.2295+2	931.4517	931.4520	3901522.0	-0.265	0(0B)	C42 H63 N10 O14		11	17	1	(K)DNTYKYK(I)
336.8622+3	1008.5721	1008.5724	1466789.6	-0.306	0(0B)	C46 H78 N11 O14		18	26	1	(K)IDGKTYLAK(Y)
504.7899+2	1008.5724	1008.5724	9351325.0	0.0126	0(0B)	C46 H78 N11 O14		18	26	1	(K)IDGKTYLAK(Y)
504.7900+2	1008.5728	1008.5724	4597659.0	0.337	0(0B)	C46 H78 N11 O14		18	26	1	(K)IDGKTYLAK(Y)
336.8625+3	1008.5728	1008.5724	2365117.8	0.393	0(0B)	C46 H78 N11 O14		18	26	1	(K)IDGKTYLAK(Y)
336.8625+3	1008.5729	1008.5724	2347151.2	0.465	0(0B)	C46 H78 N11 O14		18	26	1	(K)IDGKTYLAK(Y)
424.5470+3	1271.6265	1271.6266	5126815.0	-0.134	0(0B)	C57 H87 N14 O19		27	37	1	(K)YPSAANTKYEK(D)
424.5472+3	1271.6270	1271.6266	9344756.0	0.279	0(0B)	C57 H87 N14 O19		27	37	1	(K)YPSAANTKYEK(D)
426.5519+3	1277.6410	1277.6412	4345903.5	-0.173	0(0B)	C61 H89 N12 O18		1	10	1	(-)LEYKGEYFTK(D)
426.5521+3	1277.6417	1277.6412	1346125.9	0.339	0(0B)	C61 H89 N12 O18		1	10	1	(-)LEYKGEYFTK(D)
455.8822+3	1365.6321	1365.6321	4418268.0	-0.0367	0(0B)	C62 H89 N14 O21		5	15	1	(K)GEYFTKDNTYK(Y)

Based on the mass spectrometric data, 74% of the NFAPΔC sequence was identified (highlighted with red) from the >10 kDa fraction of the ferment broth: **LEYKGEYFTKDNTYKYKIDGKTYLAKYPSAANTKYEK**DGNKYTYDSYNRKVK**YDFRH**.

In the <10 kDa, 3-10 kDa, and <3 kDa fractions 65% of the NFAPΔC sequence was found (highlighted with red): **LEYKGEYFTKDNTYKYKIDGKTYLAKYPSAANTKYEK**DGNKYTYDSYNRKVKYDFRH.

Sites of tryptic digestion are underlined. NFAPΔC: cysteine deletion *Neosartorya fischeri* NRRL 181 antifungal protein mutant.

Table S2: Secondary structural contributions determined by the CDSSTR method from the ECD spectra of native and recombinant NFAP measured at 25 °C.

	Helix1	Helix2	Strand1	Strand2	Turns	Unordered	Total
**native NFAP**	-0.02	0.03	0.32	0.19	0.18	0.27	0.97
**recombinant NFAP**	-0.01	0.03	0.30	0.18	0.20	0.28	0.98

Native NFAP: NFAP produced by *Neosartorya fischeri* NRRL 181**; recombinant NFAP:** NFAP produced by *Pichia pastoris* KM71H.

a

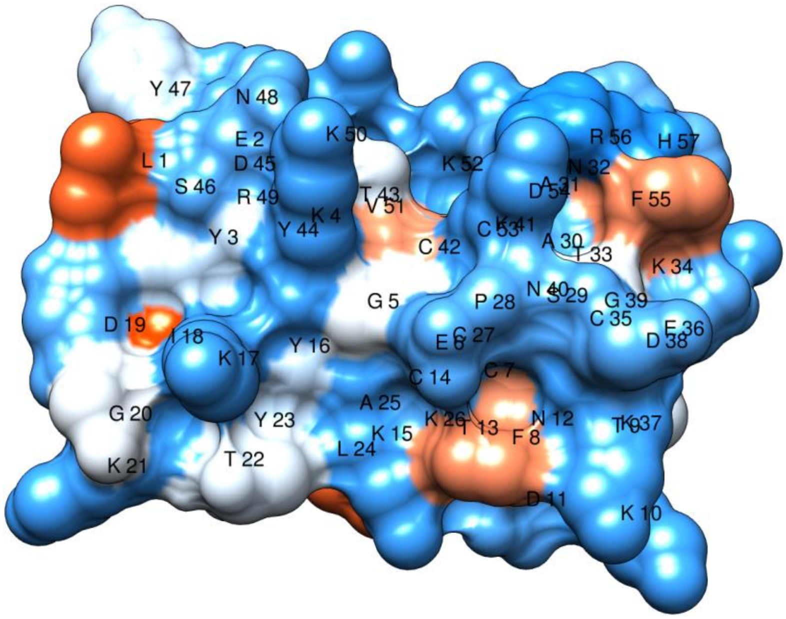

b

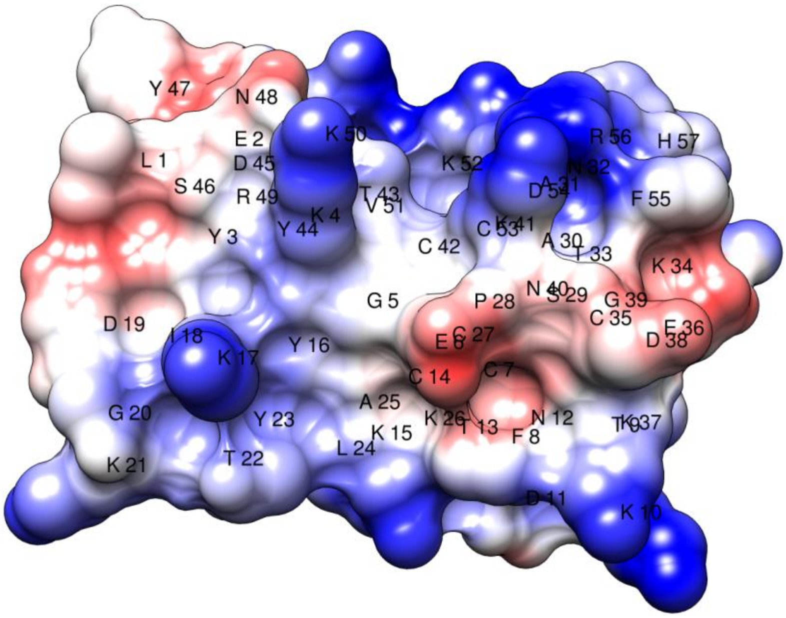

Figure S1: Hydrophobicity and electrostatic surface analyses of the predicted tertiary structure of NFAP.

The UCSF Chimera software (*Comput. Chem.* **25**, 1605-1612 (2004)) was used for the prediction. (**a**) Hydrophobicity surface analysis of NFAP, where blue and orange colours indicate the hydrophilic and hydrophobic patches, respectively. (**b**) Electrostatic surface analysis (Coulombic Surface Colouring) of NFAP, where blue and red patches indicate the positively and negatively charged patches, respectively. NFAP: *Neosartorya fischeri* NRRL 181 antifungal protein.

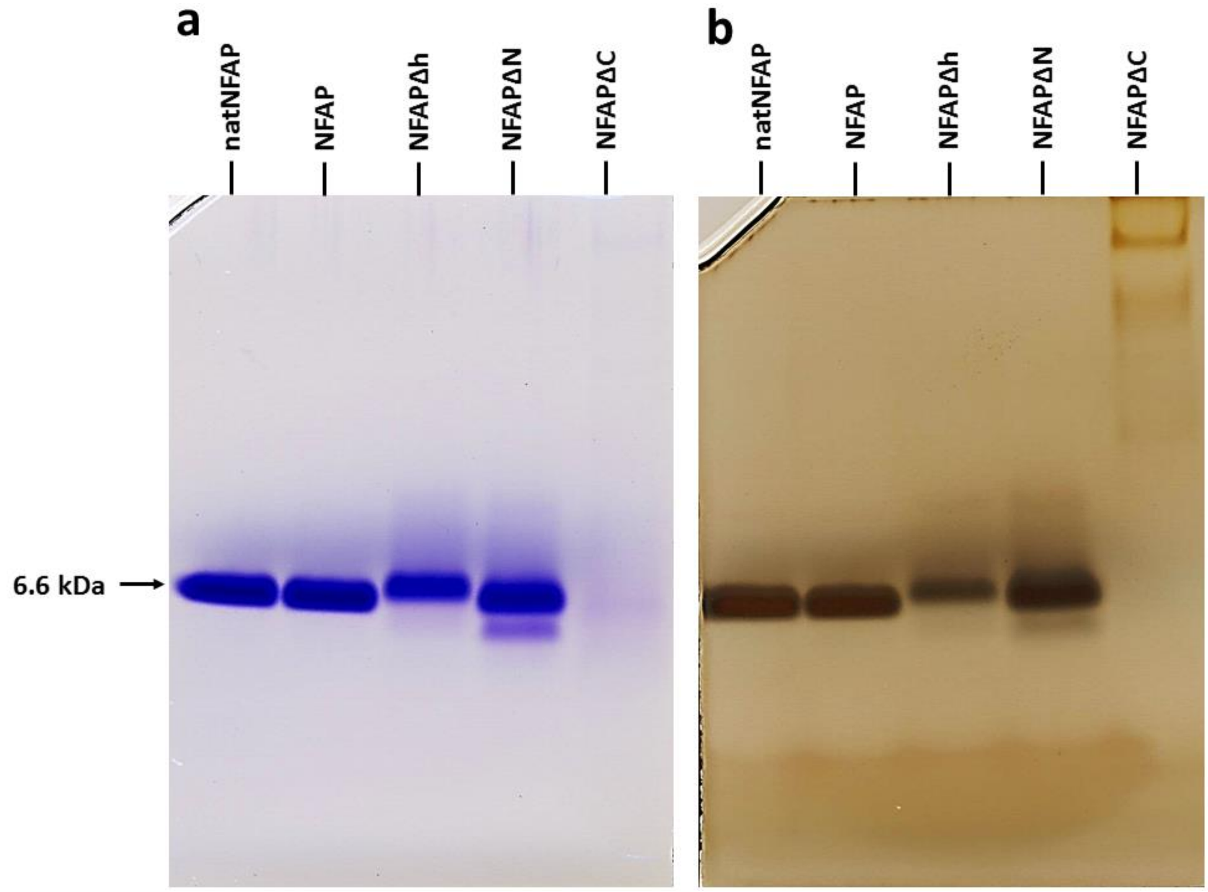

Figure S2: Appearance of the native and recombinant NFAPs in 4-12% (w/v) Bis-Tris sodium dodecyl sulphate-polyacrylamide gel.

The 4-12% (w/v) Bis-Tris sodium dodecyl sulphate-polyacrylamide gel (NuPAGE™ Novex™ 4-12% Bis-Tris Protein Gels, 1.5 mm, 10-well; Thermo Fisher Scientific, Waltham, MA, USA) in MES buffer (Thermo Fisher Scientific, Waltham, MA, USA) was stained with (**a**) Coomassie Brilliant Blue R-250 and (**b**) silver staining. natNFAP: 10 μg native *Neosartorya fischeri* NRRL 181 antifungal protein NFAP as size standard (6.6 kDa), NFAP: recombinant *Neosartorya fischeri* NRRL 181 antifungal protein; NFAPΔh: 10 μg hydrophobic core deletion NFAP mutant; NFAPΔN: 10 μg N-terminal amino acids exchanged NFAP mutant, NFAPΔC 20 μl ferment broth of recombinant cysteine deletion NFAP mutant-producer *Pichia pastoris* KM71H.

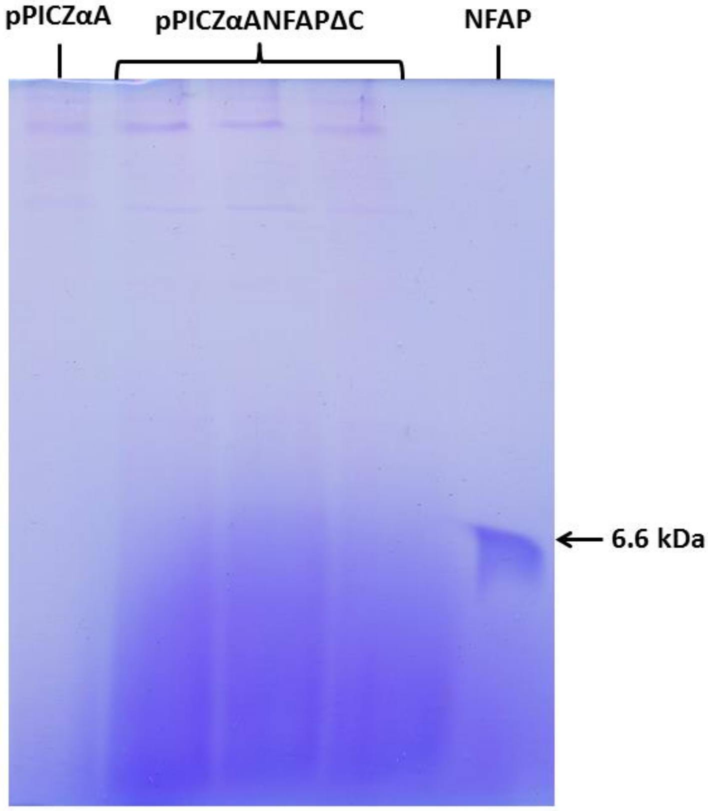

Figure S3: Comparison of the protein content of three independently produced ferment broths of NFAPΔC producing *Pichia pastoris* KM71H with the ferment broth of a mock transformed control (20 µl/lane).

The 18% (w/v) tris-glycine sodium dodecyl sulphate-polyacrylamide gel was stained with Coomassie Brilliant Blue R-250. pPICZαA: mock transformed control; pPICZαANFAPΔC: NFAPΔC producing transformant; NFAP: 1 µg purified NFAP as size standard (6.6 kDa). NFAP: recombinant *Neosartorya fischeri* NRRL 181 antifungal protein; NFAPΔC: cysteine deletion NFAP mutant.

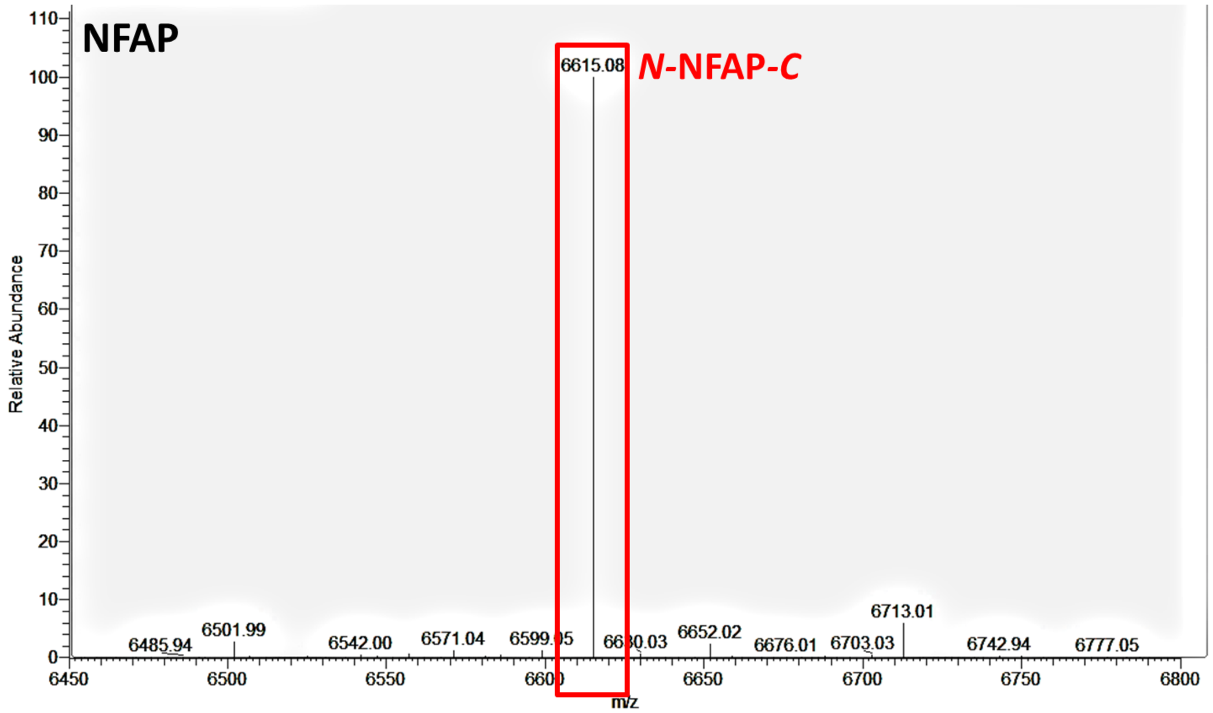
NFAP
N-NFAP-C
6615.08
6485.94
6501.99
6542.00
6571.04
6599.05
6630.03
6652.02
6676.01
6703.03
6713.01
6742.94
6777.05
Relative Abundance
m/z

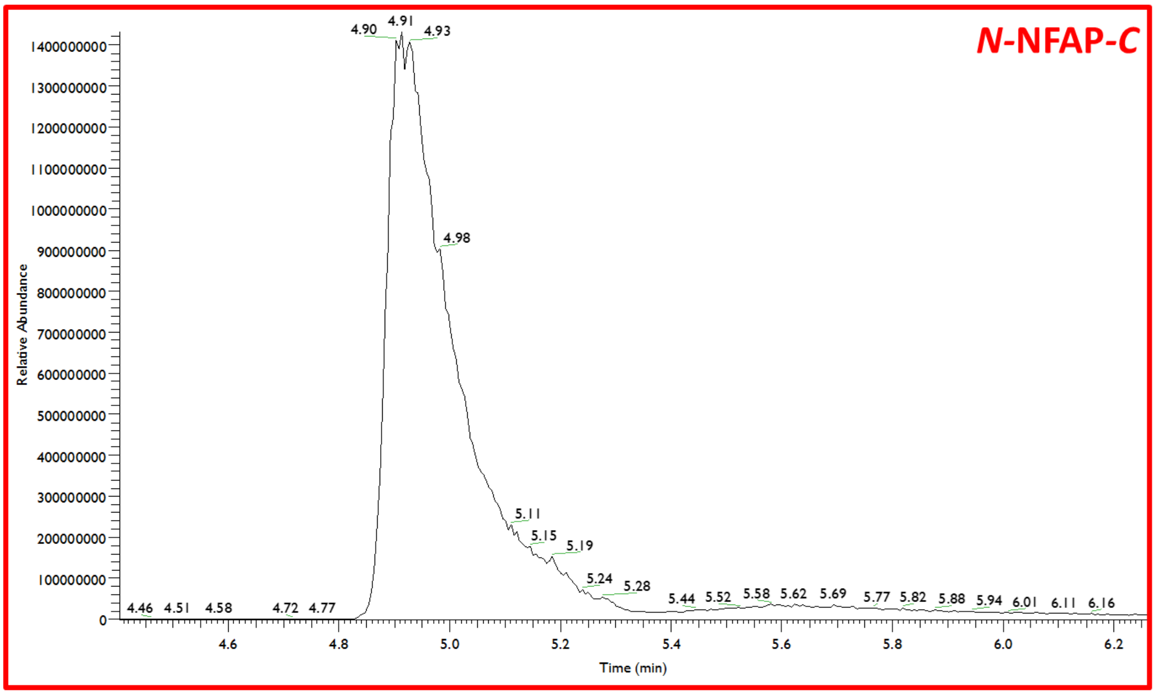
N-NFAP-C
Relative Abundance
Time (min)
4.90
4.91
4.93
4.98
5.11
5.15
5.19
5.24
5.28

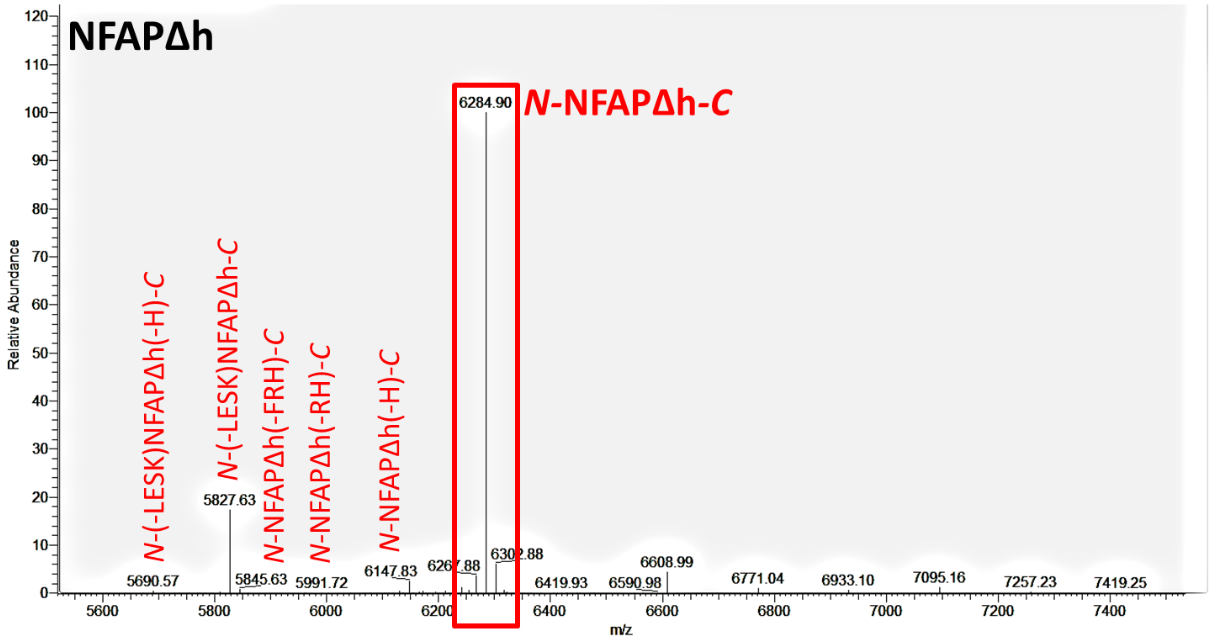
NFAPΔh
N-NFAPΔh-C
6284.90
N-(-LESK)NFAPΔh(-H)-C
N-(-LESK)NFAPΔh-C
N-NFAPΔh(-FRH)-C
N-NFAPΔh(-RH)-C
N-NFAPΔh(-H)-C
5827.63
5690.57
5845.63
5991.72
6147.83
6608.99
6419.93
6590.98
6771.04
6933.10
7095.16
7257.23
7419.25
Relative Abundance
m/z

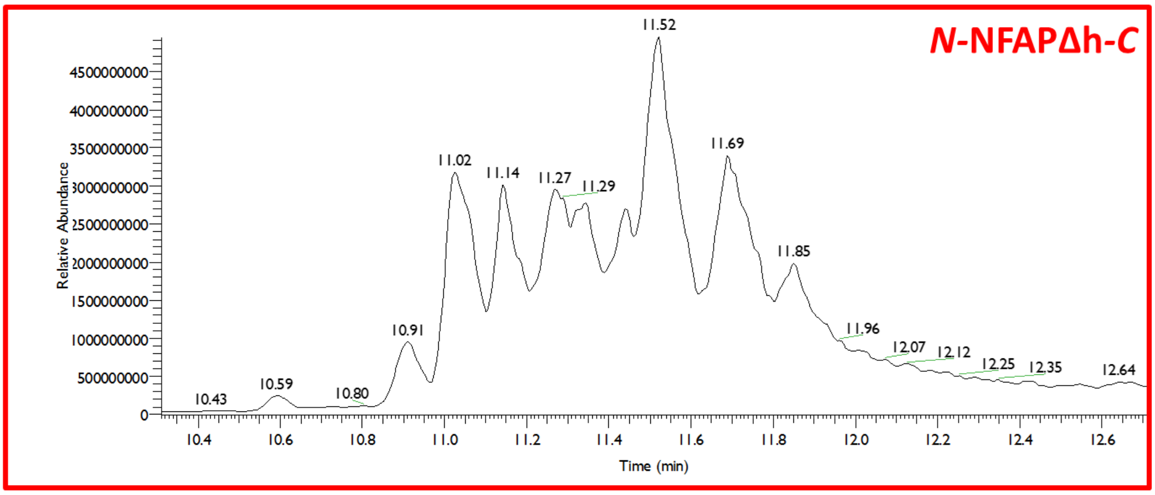
N-NFAPΔh-C
11.52
11.02
11.14
11.27
11.29
11.69
11.85
10.91
10.43
10.59
10.80
11.96
12.07
12.12
12.25
12.35
12.64
Relative Abundance
Time (min)

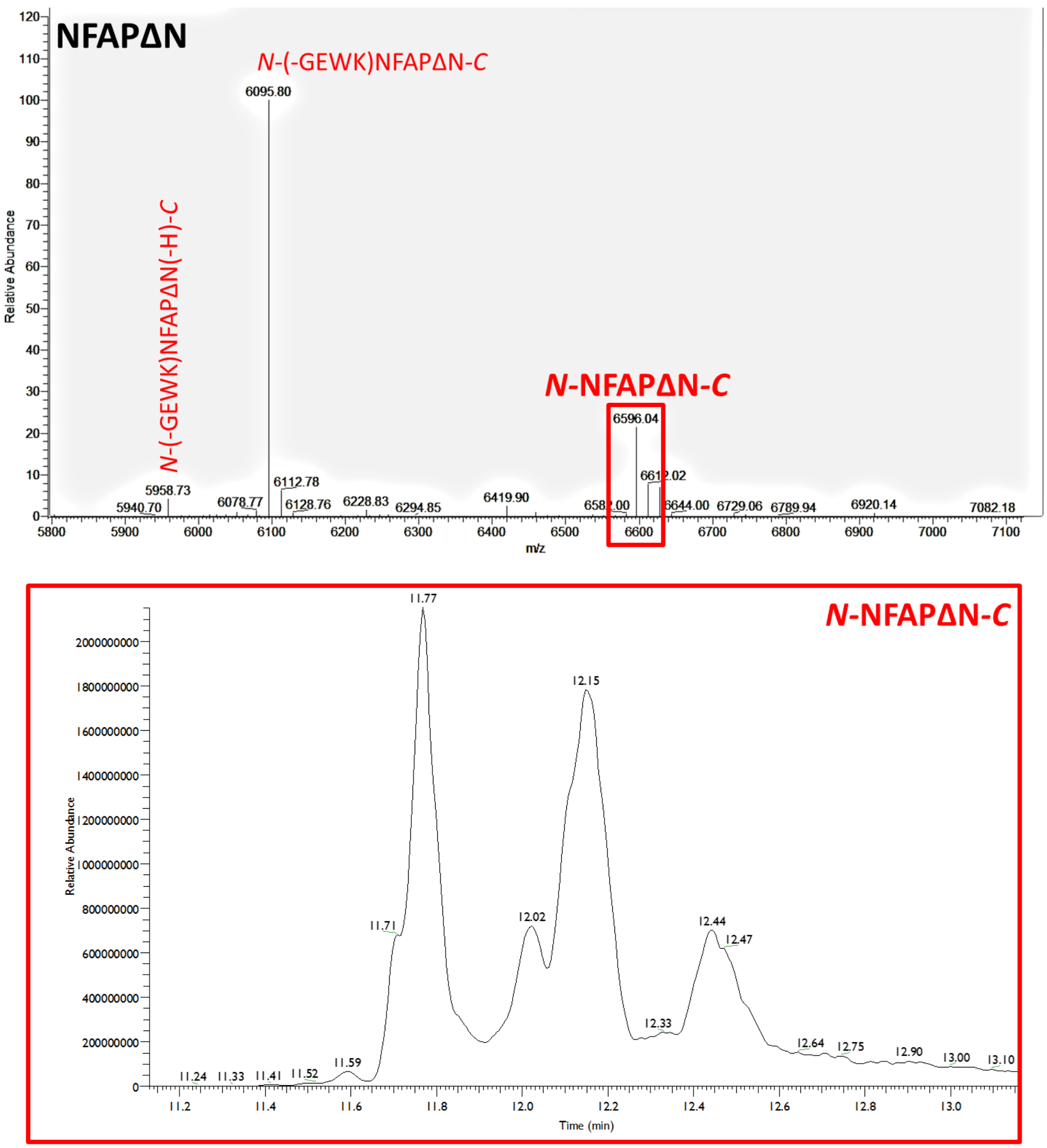

Figure S4: Monoisotopic molecular masses and mass spectrum extraction of NFAP, NFAPΔh and NFAPΔN.

Mass spectrum extraction of *N*-NFAP-*C* (Mw: 6615.08 Da), *N*-NFAPΔh-*C* (Mw: 6284.90), and *N*-NFAPΔN-*C* (Mw: 6596.04) from Table 2 are shown in red frame below the mass spectrum of the protein sample. NFAP: recombinant *Neosartorya fischeri* NRRL 181 antifungal protein; NFAPΔh: hydrophobic core deletion NFAP mutant; NFAPΔN: N-terminal amino acids exchanged NFAP mutant.

a

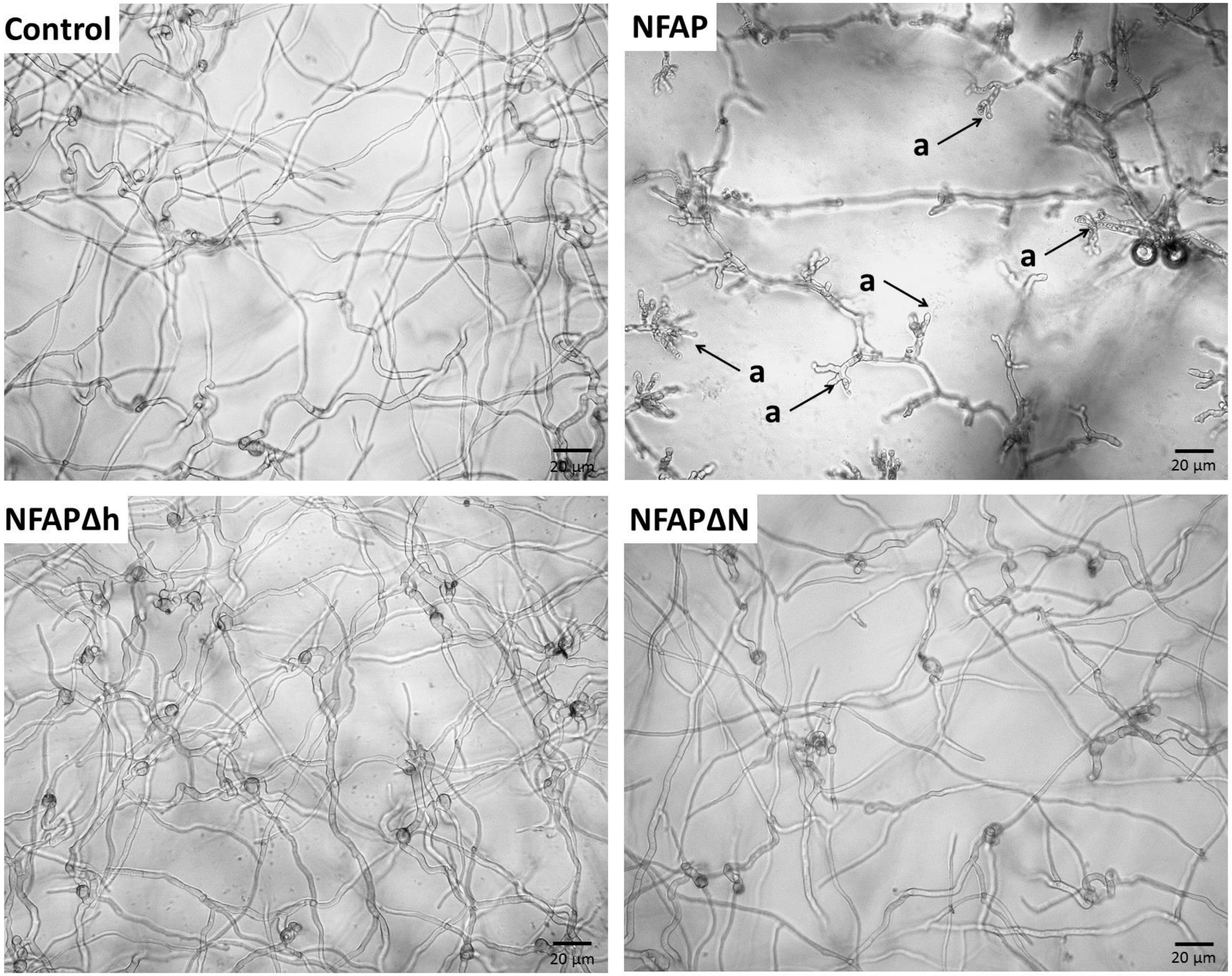

b

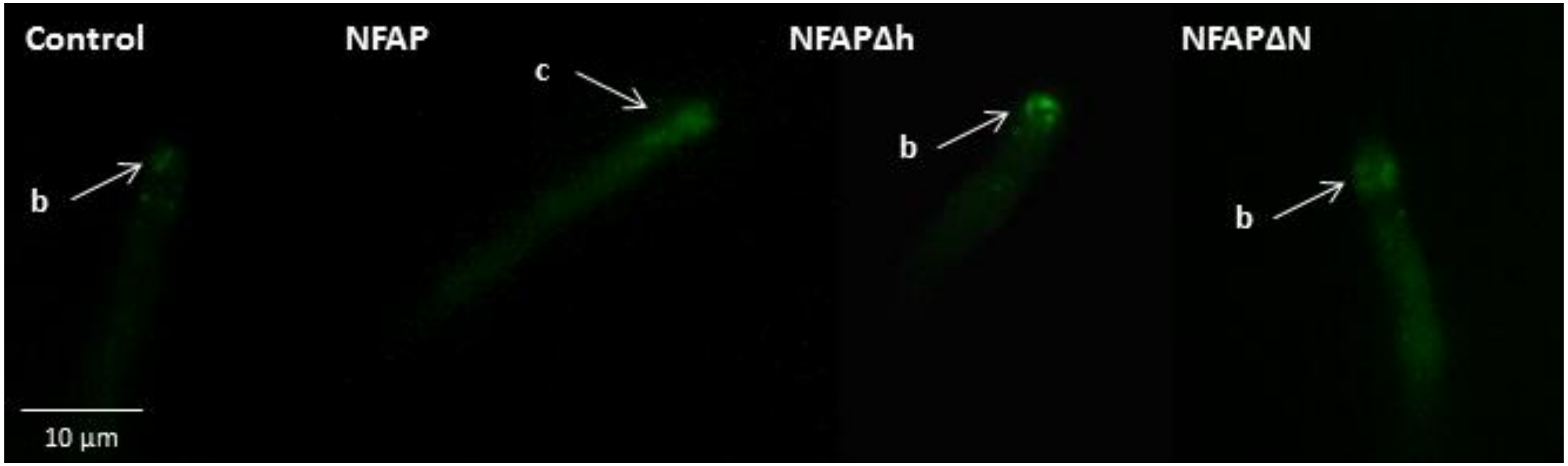
Control
NFAP
NFAPΔh
NFAPΔN
c
b
b
b
10 µm

c

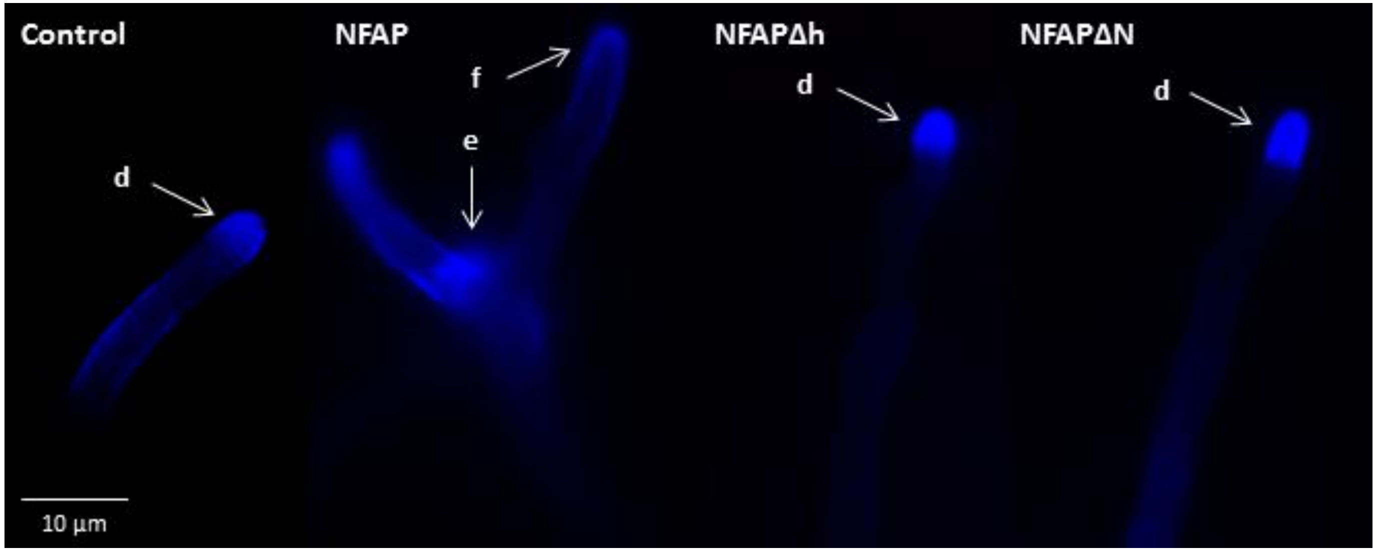
Control
NFAP
NFAPΔh
NFAPΔN
f
e
d
d
d
10 µm

d

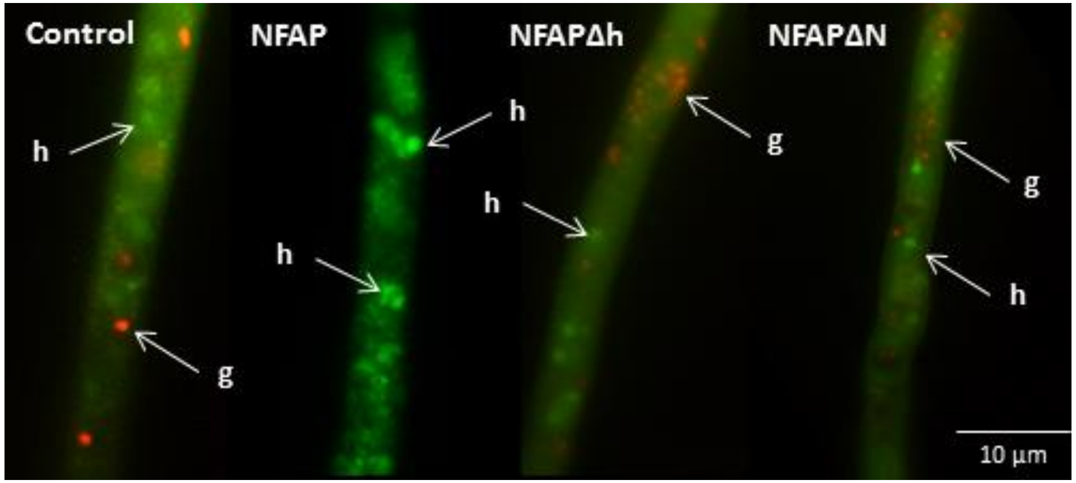

Figure S5: Morphological and physiological changes of *Aspergillus nidulans* FGSC A4 hyphae after treatment with NFAP, NFAPΔh and NFAPΔN.

(**a**) Effect of 40 µg NFAP, NFAPΔh, and NFAPΔN on the hyphal morphology of *Aspergillus nidulans* FGSC A4 after 48 h of incubation at 37 °C. a: multiple branched hyphae with swollen tips. (**b**) Actin distribution in *A. nidulans* GR5 hyphal tips in response to NFAP, NFAPΔh, and NFAPΔN treatment (25 µg/ml) for 30 min at 30 °C. b: characteristic cap/ring organized actin patches, c: distributed actin. (**c**) Calcofluor white (CFW) staining of *A. nidulans* FGSC A4 hyphae after NFAP, NFAPΔh, and NFAPΔN treatment (25 µg/ml) for 30 min at 37 °C. d: characteristic cap-like CFW fluorescence - site of the chitin assembly, e: CFW fluorescence at hyphal branching - delocalized, disturbed chitin deposition, f: lack of the characteristic cap-like CFW fluorescence. (**d**) Viability staining of *A. nidulans* FGSC A4 hyphae with FUN-1 dye after NFAP, NFAPΔh, and NFAPΔN treatment (25 µg/ml) for 30 min at 37 °C. Red vacuoles (g) indicate metabolic activity, while green vacuoles (h) show metabolic inactivity. Control: Untreated *Aspergillus nidulans* FGSC A4 or *A. nidulans* GR5 hyphae. NFAP: recombinant *Neosartorya fischeri* NRRL 181 antifungal protein; NFAPΔh: hydrophobic core deletion NFAP mutant; NFAPΔN: N-terminal amino acids exchanged NFAP mutant.

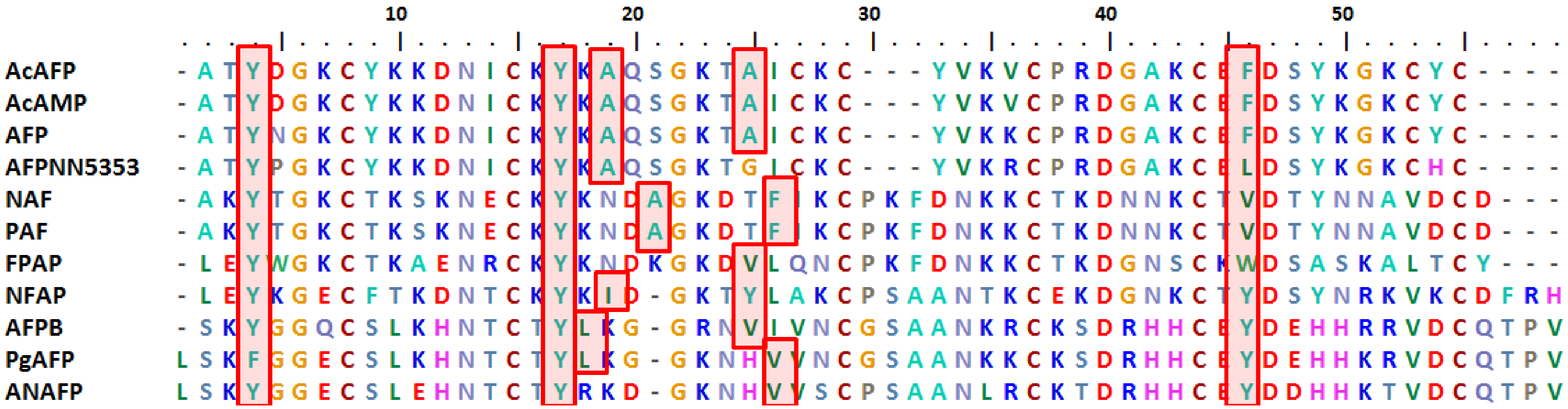

Figure S6: Alignment of the isolated and characterized NFAP-related cysteine-rich antifungal proteins from filamentous ascomycetes. The possible hydrophobic core constituting amino acids are framed and highlighted in red. Amino acids are given in colour code. AcAFP: *Aspergillus clavatus* VR1 antifungal protein (Acc. No.: A1CSS4); AcAMP: *Aspergillus clavatus* ES1 antimicrobial peptide (Acc. No.: D3Y2M3); AFP: *Aspergillus giganteus* MDH 18894 antifungal protein (Acc. No.: P17737); $\text{AFP}_{\text{NN5353}}$: *Aspergillus giganteus* A3274 antifungal protein (Acc. No.: -; *BMC Microbiol.* **11**, 209 (2011)); AFPB: *Penicillium digitatum* CECT 20796 antifungal protein (Acc. No.: K9FGI7); ANAFP: *Aspergillus niger* KCTC 2025 antifungal protein (Acc. No.: A2QM98); FPAP: *Fusarium polyphialidicum* SZMC 11042 antifungal protein (Acc. No.: E1UGX4); NAF: *Penicillium nalgiovense* BFE 66, 67, 474 antifungal protein (Acc. No.: -; *Int. J. Food. Microbiol.* **62**, 95-101 (2000)); NFAP: *Neosartorya fischeri* NRRL 181 antifungal protein (Acc. No.: D4YWE1); PAF: *Penicillium chrysogenum* Q176 antifungal protein (Acc. No.: B6HWK0); PgAFP: *Penicillium chrysogenum* RP42C antifungal protein (Acc. No.: D0EXD3).
